# Supplementary material for: DEAD-box ATPase Dbp2 is the key enzyme in an mRNP assembly checkpoint at the 3’-end of genes and involved in the recycling of cleavage factors
Source: Nat Commun. 2024 Aug 9;15:6829. doi: 10.1038/s41467-024-51035-z (PMC11315920; doi:10.1038/s41467-024-51035-z)
Supplement: Supplementary file 1 — Supplementary Information [file 41467_2024_51035_MOESM1_ESM.pdf]

Supplementary Figures

Suppl. Figure 1

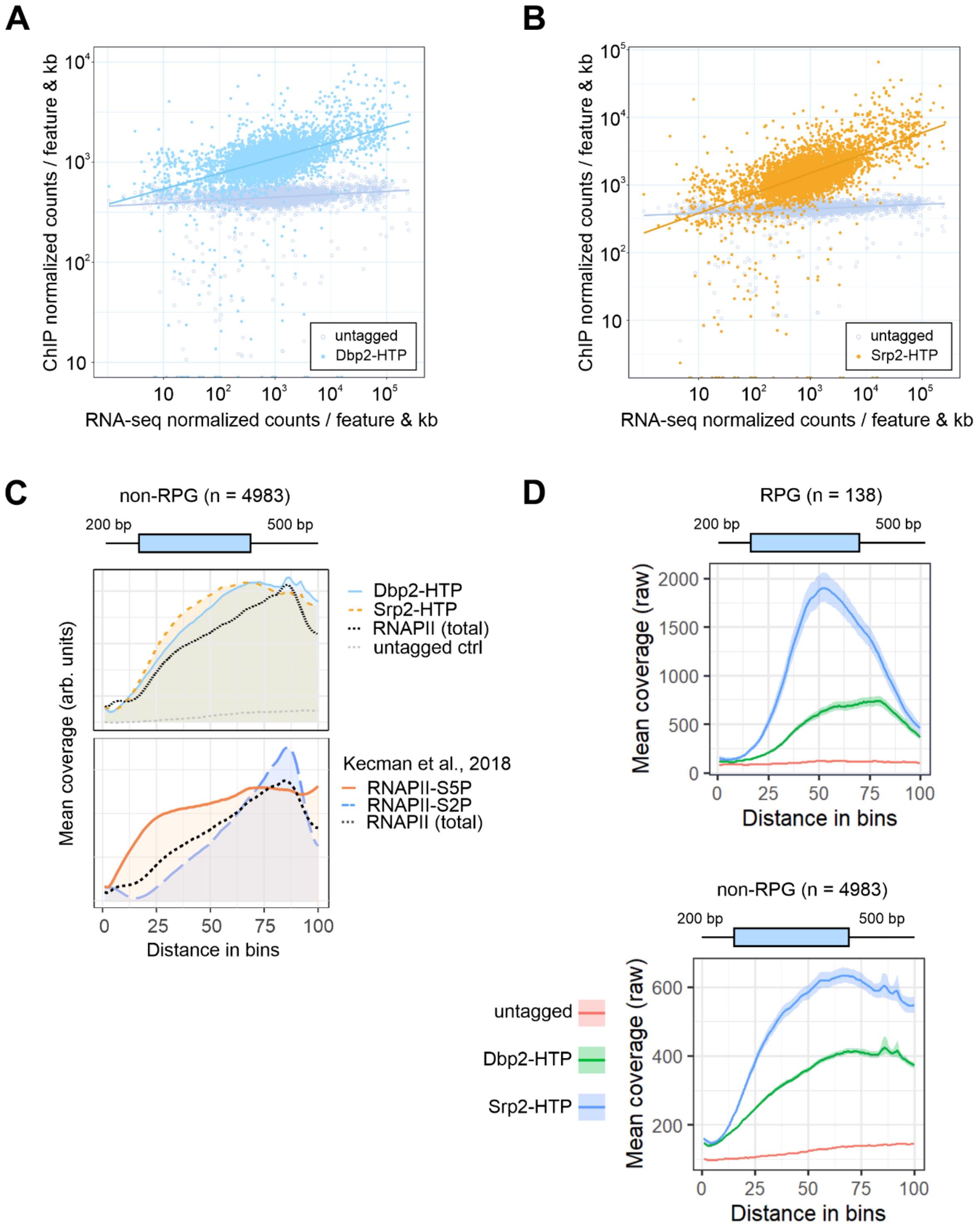

Supplementary Figure 1

A and B Integrated counts of Dbp2-HTP (A) and Srp2-HTP (B) ChIP-seq signal across protein-coding genes relative to RNA-seq expression levels in the wild type, given as average counts per feature and kb * 1,000,000 (n = 3 biological replicates). ChIP in the untagged wild type was included as control. Trendlines were fitted using linear regression. RNA-seq expression data is from the untagged isogenic wild type, given as average counts per feature and kb * 1,000,000 (n = 3 biological replicates). Source data are provided as a Source Data file.

C Metagene analysis of mean ChIP-seq coverage of Dbp2-HTP, Srp2-HTP and an untagged control (IgG-ChIP; n = 3 biological replicates), and total RNAPII (α-rpb1 (8WG16) in Dbp2-3myc; n = 2) across transcription start sites and the annotated 3'-ends of genes for all protein-coding genes excluding ribosomal protein genes (n = 4983 genes) (upper panel). Total Rpb1, Rpb1-S5P, and Rpb1-S2P ChIP for an isogenic wild type (n = 2 biological replicates) are included as reference (lower panel; data from Kecman et al., 2018; GEO: GSE111326). Genes with flanking regions that exceed the chromosome boundaries were excluded from the analysis. Mean coverage given in arbitrary units; to compensate for differences in ChIP capability, mean coverage was adjusted by a constant scaling factor for an easier comparison of curve shapes. Non-scaled curves with confidence intervals are provided in Suppl. Figure 1D.

D Non-scaled curves with confidence intervals for the metagene analysis in Figure 1C and S1C, showing metagene analyses of mean ChIP-seq coverage of Dbp2-HTP, Srp2-HTP and an untagged wild-type control (IgG-ChIP, n = 3 biological replicates) across ribosomal protein genes (RPGs; n = 138 genes) including 200 bp upstream and 500 bp downstream of the annotated transcription units (upper panel) or all protein-coding genes excluding RPGs (non-RPGs; n = 4983 genes) (lower panel). Genes with flanking regions that exceed the chromosome boundaries were excluded from the analysis.

Suppl. Figure 2

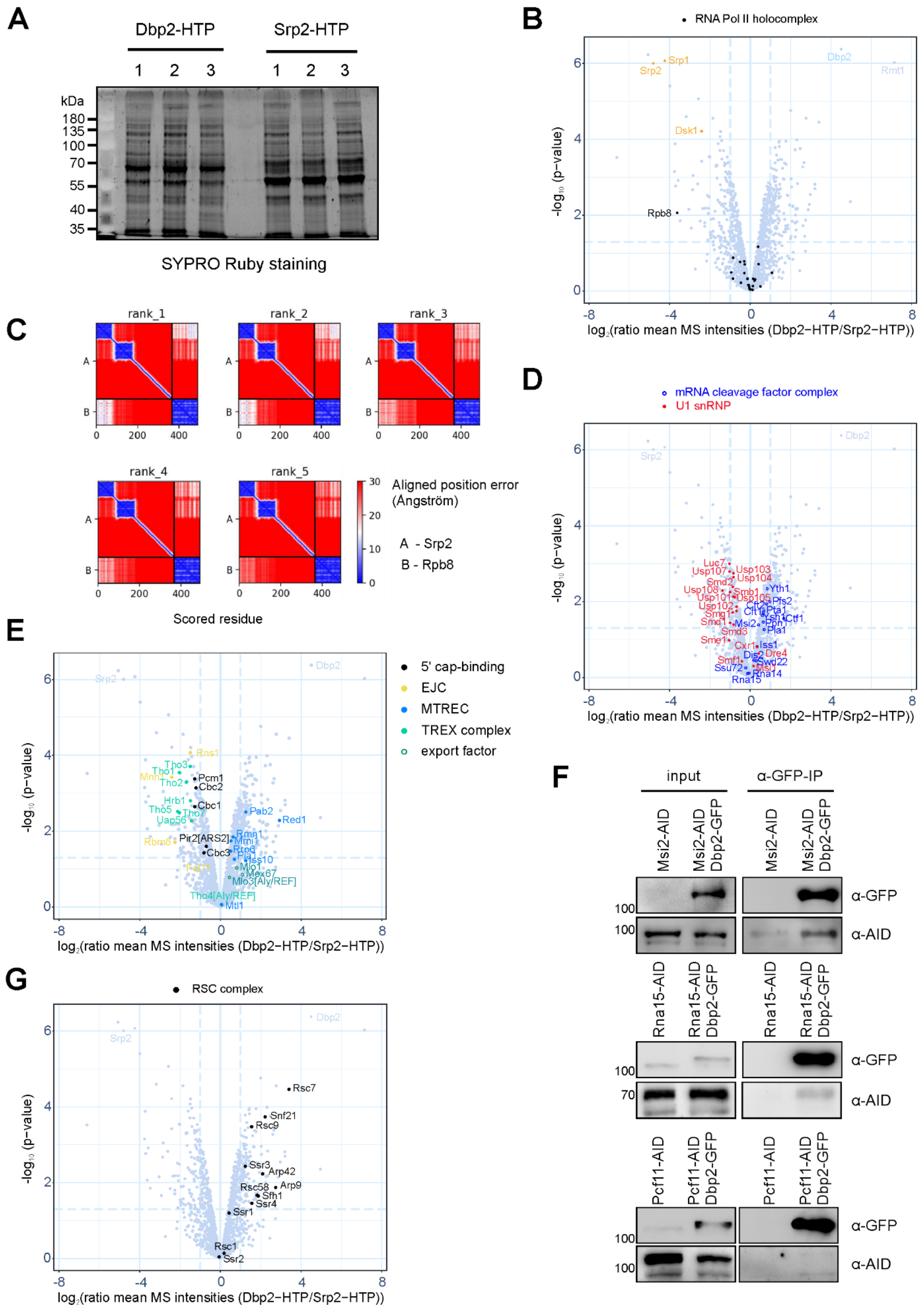

Supplementary Figure 2

A Eluates of cross-linking HTP purifications of Dbp2-HTP and Srp2-HTP were resolved on SDS-PAGE and stained with SYPRO Ruby. The experiment was carried out with n = 3 biological replicates.

B Mass spectrometry (MS) analysis of the comparative interaction profiling of Dbp2 and Srp2. In the volcano plot, p-values ($-\log_{10}$, two-sided moderated Student's t-test) are plotted against the relative enrichment of proteins in the purification of Dbp2-HTP relative to Srp2-HTP based on mean protein intensities ($\log_2$) (n=3 biological replicates). Components of the RNAPII holocomplex (GO:0016591) are marked in black, Srp2 and known interactors in orange, and Dbp2 light blue. Dashed horizontal line indicates a p-value of 0.05, dashed horizontal lines 2-fold enrichment. Source data are provided as a Source Data file.

C AlphaFold2-multimer prediction of Srp2-Rpb8 interaction, visualised using predicted aligned error[1]. The interaction is predicted by all five default Alphafold2-multimer models, ranked by the confidence with which they predict the interaction. Blue and white shades in the interaction quadrants (upper right and lower left) denote low expected position error, indicative of a protein-protein interaction[2]. Srp2: x-axis amino acid residues 1-365 and y-axis protein A; Rpb8: x-axis amino acid residues 366-490 and y-axis protein B.

D MS analysis of the comparative interaction profiling of Dbp2 and Srp2 as in A. Components of the mRNA cleavage factor complex (GO:0005849) and U1 snRNP (GO:0005685) are labelled in blue and red, respectively. Source data are provided as a Source Data file.

E MS analysis of the comparative interaction profiling of Dbp2 and Srp2 as in A. Known nuclear cap-interacting proteins, components of the exon junction complex (EJC), the Mtl1-Red1 core (MTREC), the TREX complex and selected export factors are labelled in black, orange, blue, green, and dark green, respectively. Source data are provided as a Source Data file.

F Lysates and eluates of a co-immunoprecipitation with GFP-Trap beads from strains expressing either Msi2-AID, Pcf11-AID or Rna15-AID in the presence or absence of GFP-tagged Dbp2 were resolved on SDS-PAGE and analysed by Western blot against GFP and the AID tag. The AID-tagged strains in which the bait protein carries no tag are included as control for background binding to the beads. Images are representative of two independent experiments. Uncropped images are provided in the Source Data file.

G MS analysis of the comparative interaction profiling of Dbp2 and Srp2 as in A. Components of the RSC complex (GO:0016586) are labelled in black. Source data are provided as a Source Data file.

Suppl. Figure 3

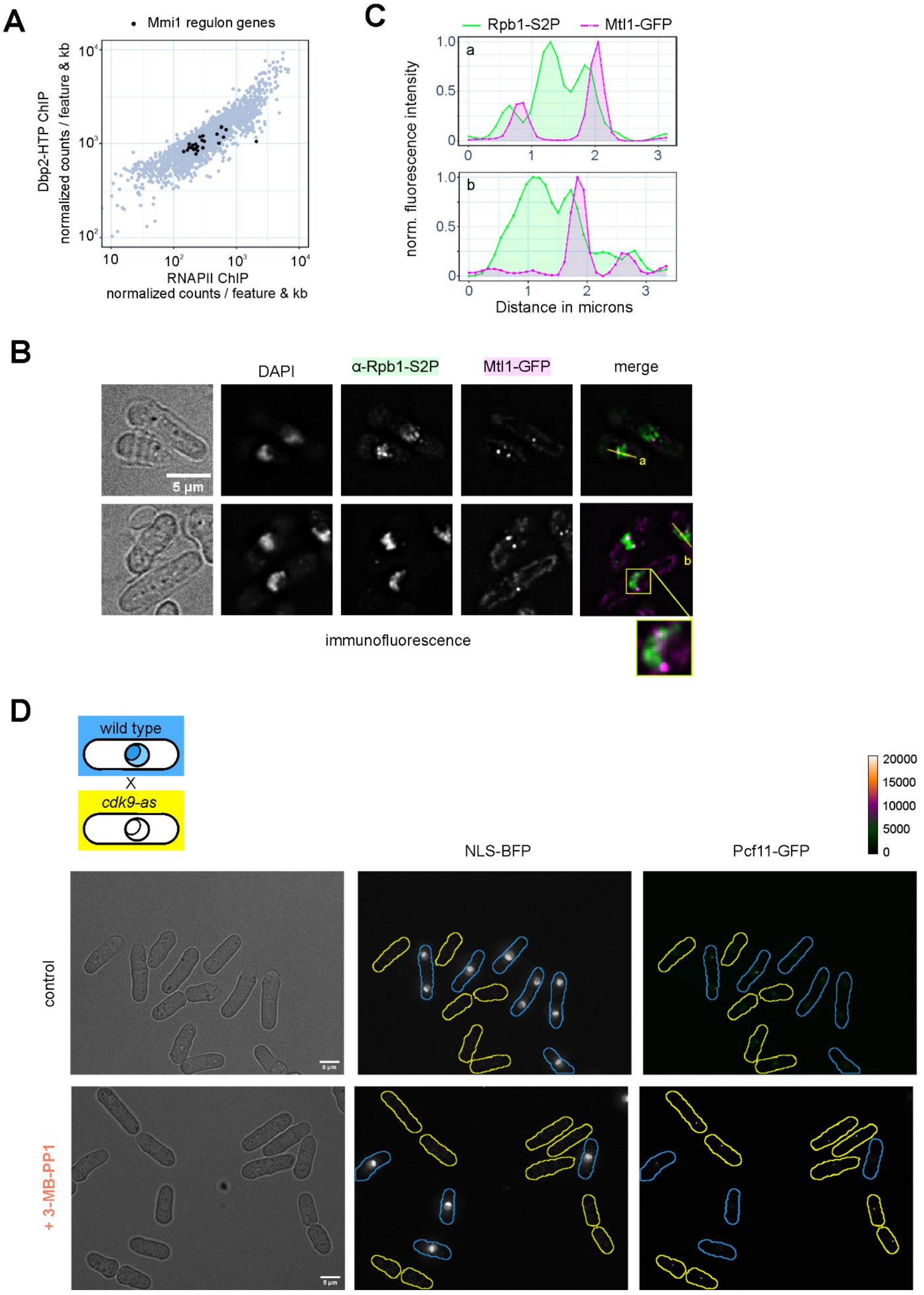

Suppl. Figure 3E

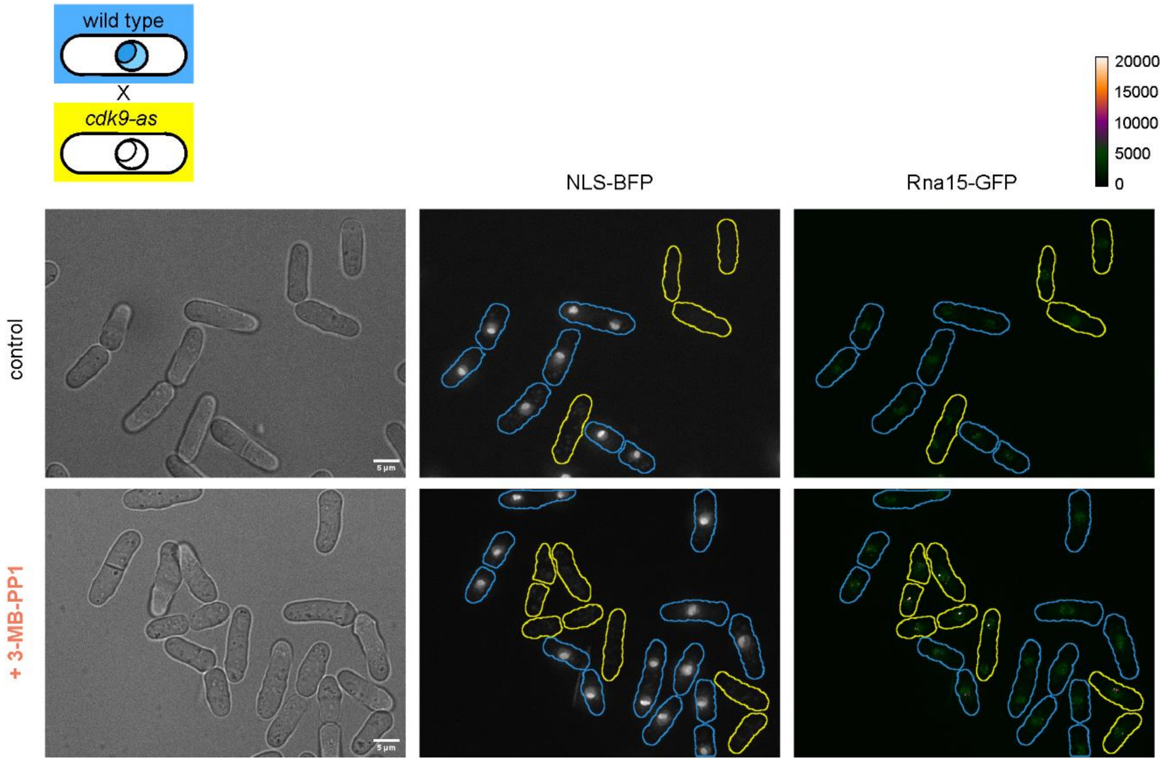

Supplementary Figure 3

A Integrated counts of Dbp2-HTP ChIP-seq signal across protein-coding genes relative to RNAPII, given as average counts per feature and kb * 1,000,000 (n = 3 biological replicates) as in 1B. Only genes with an RNAPII occupancy above a set threshold of 10 normalized counts per feature and kb per million were included. RNAPII ChIP-seq data from Kecman et al., 2018; GEO: GSE111326. Genes that belong to the Mmi1 regulon[3] are marked in black. Source data are provided as a Source Data file.

B Immunofluorescence against Rbp1-S2P in a strain where MTREC component Mtl1 was genomically tagged with GFP. Cells were grown in YES at 30°C and fixed with formaldehyde directly in the growth medium. Rpb1-S2P was detected with anti-RNA polymerase II CTD repeat YSPTSPS (phospho S2) (Abcam, ab252855, 1:1000). The merged channel shows Rpb1-S2P in green and Mtl1-GFP in magenta. Fluorescence intensity profiles were generated along the yellow lines and are shown in C. Images are representative of two independent experiments.

C Fluorescence intensity profiles across cleavage bodies and sites of active transcription as indicated in B. Green line corresponds to the Rpb1-S2P signal, dashed magenta line to the residual Mtl1-GFP signal. Fluorescence intensities were normalised to a 0-1 range. Source data are provided as a Source Data file.

D Live cell imaging of mixed cultures of wild type (marked by NLS-BFP; blue outlines) and an analog-sensitive mutant of Cdk9 (*cdk9-as*; yellow outlines) with Pcf11 tagged with GFP. Cells were grown in in YES at 30°C and treated with 100 µM 3-MB-PP1 for 10 min to block transcription elongation before pelleting and resuspension in EMMG + 3-MB-PP1 for imaging (lower panels). Non-treated cells were included as control (upper panels). The GFP channel is shown as the maximal intensity projection using a blue orange icb colour scale as indicated. Images are representative of two independent experiments.

E Live cell imaging of mixed cultures of wild type (marked by NLS-BFP; blue outlines) and an analog-sensitive mutant of Cdk9 (*cdk9-as*; yellow outlines) with Rna15 tagged with GFP. Cells were grown in in YES at 30°C and treated with 100 µM 3-MB-PP1 for 10 min to block transcription elongation before pelleting and resuspension in EMMG + 3-MB-PP1 for imaging (lower panels). Non-treated cells were included as control (upper panels). The GFP channel is shown as the maximal intensity projection using a blue orange icb colour scale as indicated. Images are representative of two independent experiments.

Suppl. Figure 4

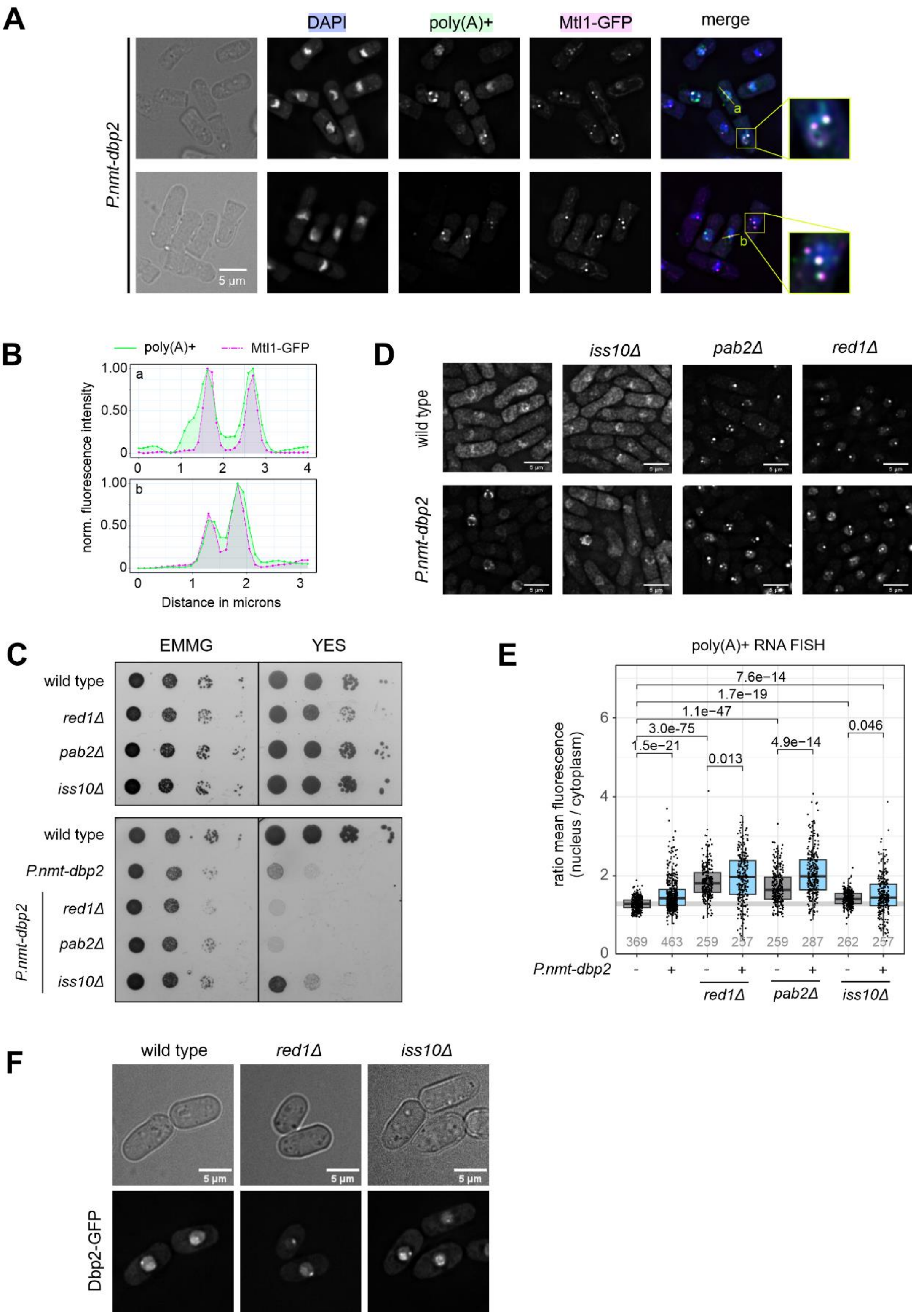

Supplementary Figure 4

A FISH-IF against poly(A)+ RNA using oligo-d(T)-Cy3 in *P.nmt-dbp2* with the cleavage body marker Mtl1 genomically tagged with GFP. Cells were grown overnight in EMMG, then shifted to YES for 5h to shut off the *nmt1* promoter before formaldehyde fixation. The merged channel shows poly(A)+ RNA in green, Mtl1-GFP in magenta and DAPI in blue. Fluorescence intensity profiles were generated along the yellow lines and are shown in B. Images are representative of three independent experiments. Note that in the Mtl1-GFP background, the poly(A)+ RNA signal tends to be more focused than in the untagged strain.

B Fluorescence intensity profiles of FISH-IF signal across sites of poly(A)+ RNA accumulation and the adjacent nuclear area as indicated in A. Green line corresponds to the poly(A)+ RNA signal, dashed magenta line to the Mtl1-GFP signal. Fluorescence intensities were normalised to a 0-1 range. Source data are provided as a Source Data file.

C Growth assay of MTREC mutants combined with *P.nmt-dbp2*. The indicated strains were grown in EMMG overnight, and serial dilutions (1:10) were spotted on EMMG or YES and incubated at 30°C. Images are representative of two independent experiments.

D poly(A)+ RNA FISH experiment of MTREC mutants combined with *P.nmt-dbp2* as in Figure 4C. Images show the signal of the Cy3-labelled oligo-d(T) probe and are representative of three independent experiments. A quantitation of the nuclear/cytoplasmic signal distribution is provided in E.

E Quantitation of the poly(A)+ RNA FISH experiment shown in D as in Figure 4D (n = 3 independent experiments). Semi-automated cellular segmentation was carried out by thresholding on the transmitted light channel (cell outlines) and DAPI stain (nuclei). Measurements were performed on average intensity Z-projections for nucleus and cytoplasm (= total cell without nucleus) and the ratio of mean nuclear fluorescence intensity over mean cytoplasmic fluorescence intensity calculated for each cell. Numbers below the graph indicate number of counted cells for each condition. The lower and upper hinges correspond to the first and third quartiles, and the whiskers extend from the hinge to the smallest and largest value no further than 1.5 * IQR from the hinge (where IQR is the inter-quartile range). The horizontal line marks the median value. The displayed p-values for the pair-wise comparisons were calculated using a two-sided Wilcoxon rank sum test. At least a hundred cells were counted for each condition. Source data are provided as a Source Data file.

F Live cell imaging of genomically tagged Dbp2-GFP in wild type, *red1Δ*, or *iss10Δ* as in A. Images are representative of two independent experiments.

Suppl. Figure 5

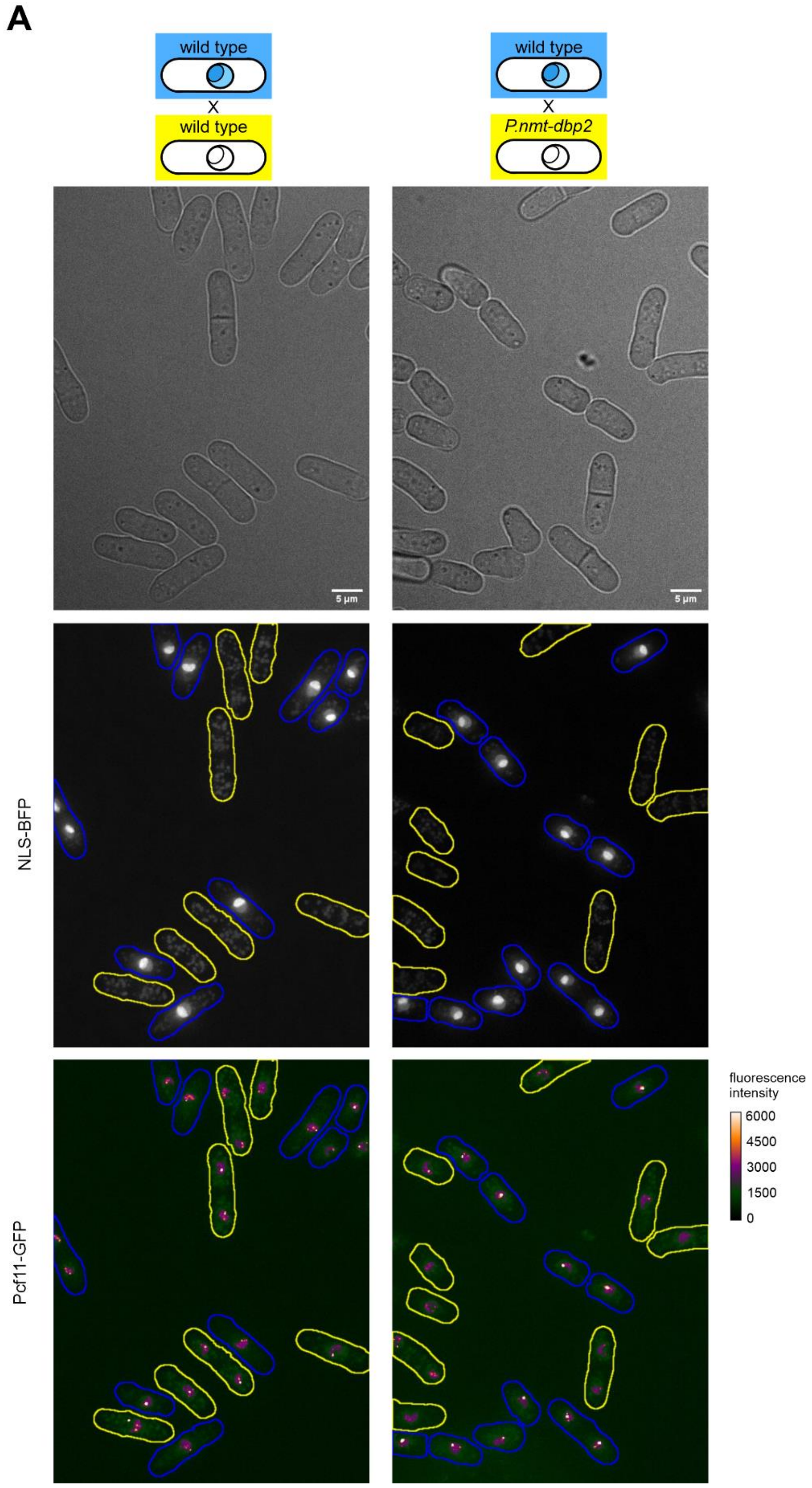

Supplementary Figure 5A

Live cell imaging of mixed cultures of wild type (marked by NLS-BFP) and *P.nmt-dbp2* with Pcf11 tagged with GFP as in Figure 5A. Cells were grown in EMMG at 30°C and shifted to YES for 6h. The GFP channel is shown as the maximal intensity projection using a blue orange icb colour scale as indicated. Cell outlines are coloured according to presence or absence of the NLS-BFP marker (+ blue; - yellow). Images are representative of two independent experiments.

Suppl. Figure 5

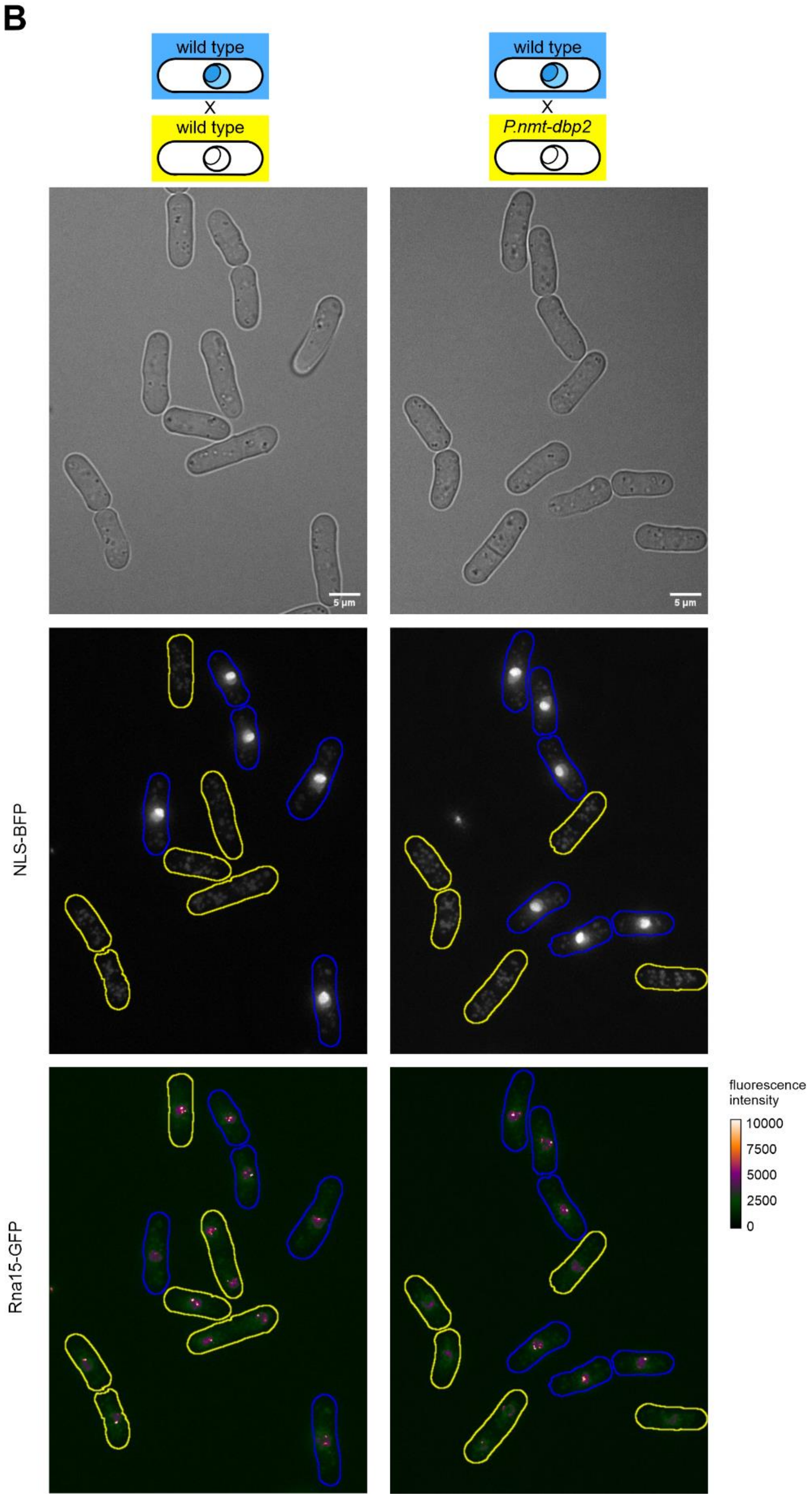

Supplementary Figure 5B

Live cell imaging of mixed cultures of wild type (marked by NLS-BFP) and *P.nmt-dbp2* with Rna15 tagged with GFP as in Figure 5A. Cells were grown in EMMG at 30°C and shifted to YES for 6h. The GFP channel is shown as the maximal intensity projection using a blue orange icb colour scale as indicated. Cell outlines are coloured according to presence or absence of the NLS-BFP marker (+ blue; - yellow). Images are representative of two independent experiments.

Suppl. Figure 6

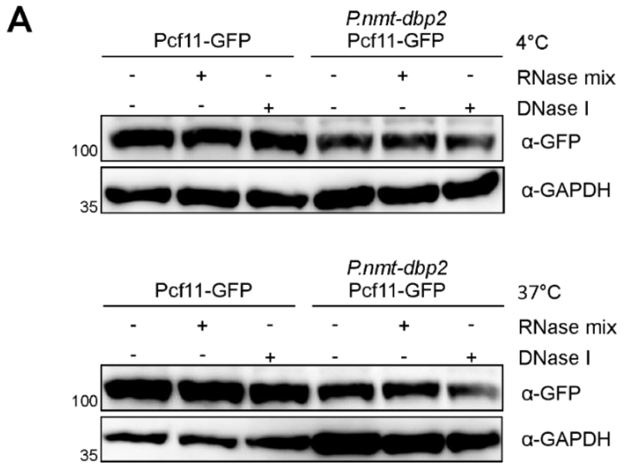

Supplementary Figure 6

A Western blot analysis of SDS lysates that were incubated with RNases or DNase for either 30 min at 4°C (top panel) or 15 min at 37°C (bottom panel) prior to the clearing spin to release RNA- or DNA-associated proteins into the soluble pool as in Figure 6C. Lysates incubated without added enzymes were included as control. The numbers on the left indicate the molecular weight marker in kDa. Images are representative of two independent experiments.

Suppl. Figure 7

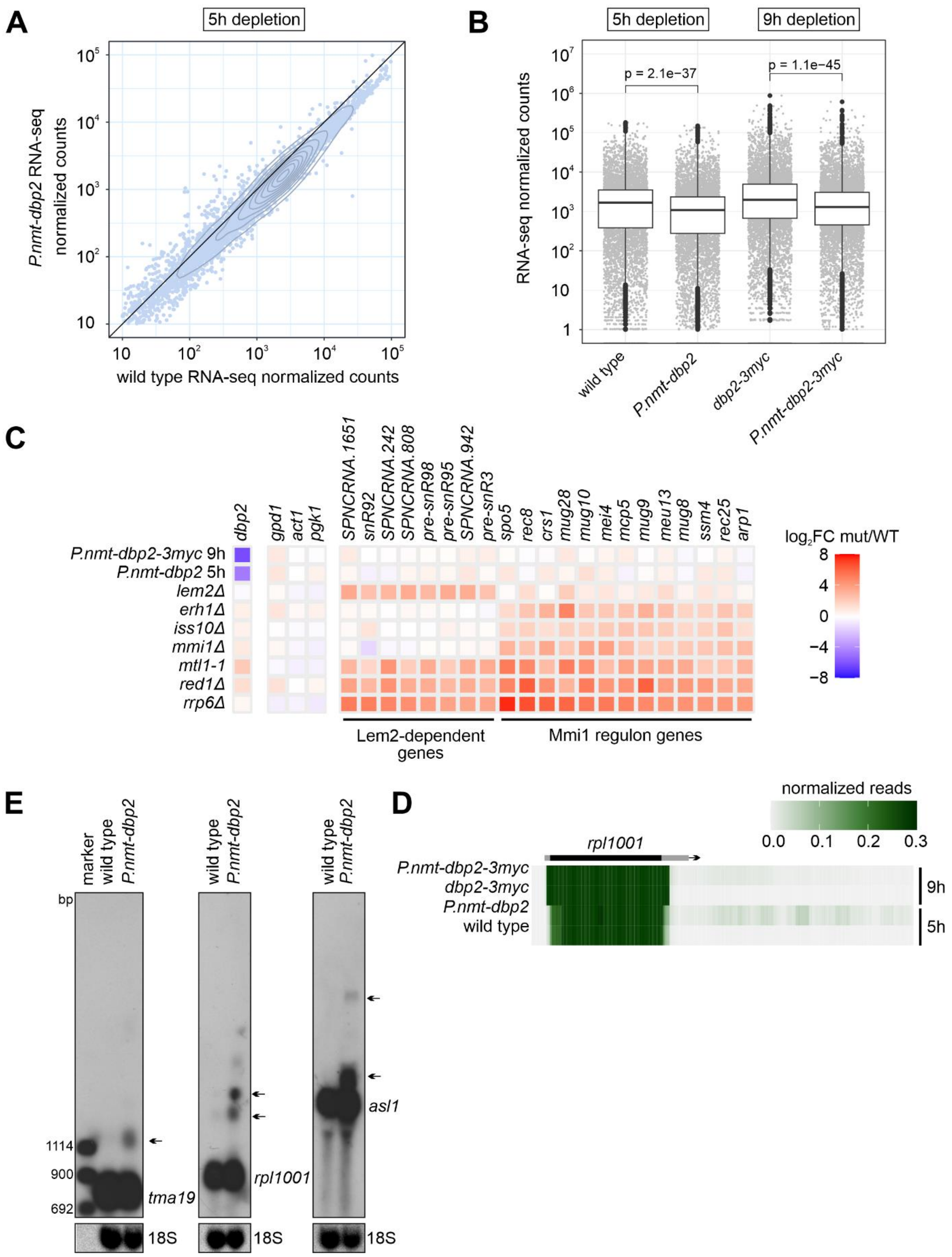

Supplementary Figure 7

A Mean integrated counts of RNA-seq reads over annotated features in wild-type and *P.nmt-dbp2* (n = 3 biological replicates) after a 5h depletion period. Cells were grown in EMMG overnight at 30°C, harvested, and cultured in YES for 5h, then mixed with *S. cerevisiae* in a 5:1 OD_{600} ratio prior to RNA isolation. Integrated counts were normalized to the number of total *S. cerevisiae* reads for each sample before calculating the mean. Contours of a 2d density estimate are shown in dark grey. Source data are provided as a Source Data file.

B Mean integrated counts of RNA-seq reads over annotated features in untagged (n = 3 biological replicates) or 3myc-tagged (n = 2 biological replicates) Dbp2 after a 5h or 9h depletion period, respectively. 5h samples were poly(A)-selected, 9h samples were ribodepleted to enrich mRNAs. Normalized counts were generated as in A. The lower and upper hinges correspond to the first and third quartiles, and the whiskers extend from the hinge to the smallest and largest value no further than 1.5 * IQR from the hinge (where IQR is the inter-quartile range). The horizontal line marks the median value. The displayed p-values for the pair-wise comparisons were calculated using a two-sided Wilcoxon rank sum test. Source data are provided as a Source Data file.

C DESeq2 differential expression analysis of nuclear exosome target genes that are either Lem2-dependent or part of the Mmi1 regulon[3,4]; *adh1*, *pgk1* and *act1* are housekeeping mRNAs and included as controls. Colour indicates $\log_2$ fold change of normalized RNA-seq counts of mutant over wild type. MTREC mutant data from [5] (*rrp6Δ*), [6] (*mmi1Δ*), [7] (*mtl1-1*), and [4] (*red1Δ, iss10Δ, erh1Δ, lem2Δ)*; Accessions PRJEB7403, GSE73144, GSE148799, and GSE174347. *S. cerevisiae* spike-in was disregarded for the analysis to obtain comparable relative expression data. Source data are provided as a Source Data file.

D Heat map of RNA-seq reads across *rpl1001* and downstream regions in untagged (n = 3 biological replicates) or 3myc-tagged (n = 2 biological replicates) Dbp2 after a 5h or 9h depletion period, respectively. Reads were counted across 10 bp bins, and the means normalized to a 0-1 range and plotted at y_{max} = 0.3 to visualize relative amounts of 3'-extended transcripts. The annotated position of the transcripts is indicated in grey, the coding sequence is highlighted in black.

E Northern blot for *tma19*, *asl1*, and *rpl1001* mRNA using a strand-specific DIG-labelled RNA probe against the gene body. Cells were grown in EMMG overnight at 30°C, harvested, and cultured in YES medium for 5h prior to RNA isolation. 18S band stained with methylene blue is shown as a loading control. Arrows indicate positions of extended transcripts. The marker is a double-stranded DNA size marker (Roche, Molecular Weight Marker VIII, DIG-labeled).

Suppl. Figure 8

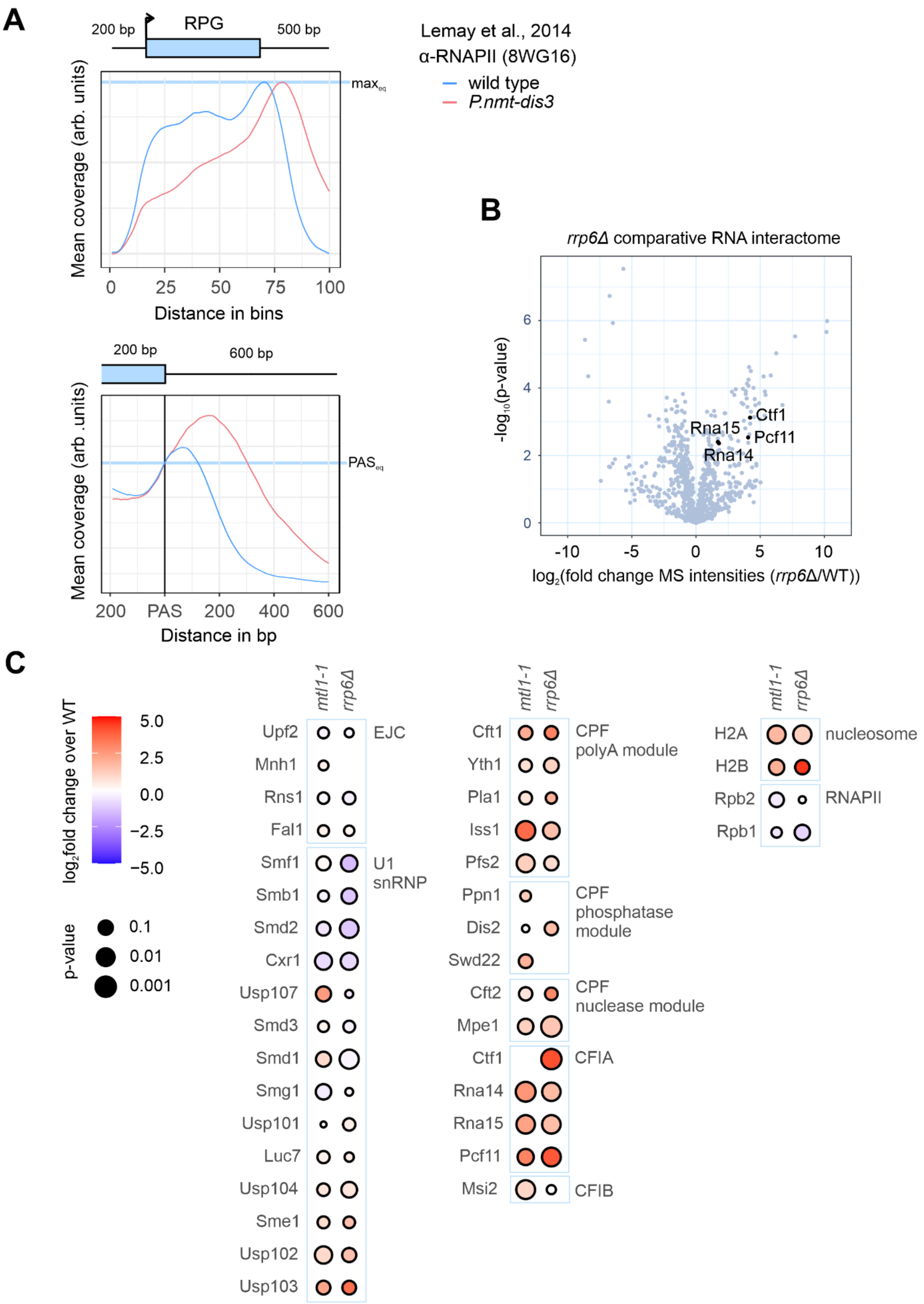

Supplementary Figure 8

A Metagene analysis of mean RNAPII ChIP-seq signal as in 7C for wild type and *P.nmt-dis3* (n = 2 biological replicates) across ribosomal protein genes (RPGs) including 200 bp upstream and 500 bp downstream of the annotated transcription units (upper panel) or surrounding the polyadenylation and cleavage site (PAS) (lower panel). Mean coverage was adjusted by a constant scaling factor to normalize to maximal peak height (upper panel) or RNAPII levels at the PAS (lower panel) for an easier comparison of curve shapes. Schematic of the gene above the left panel corresponds to an RPG of median length. Within the metagene, the positions of transcription start and end sites are distributed around the given coordinate because of the varying feature compression depending on gene length. Data from [8], ArrayExpress accession E-MTAB-2237; cells were grown in EMMG and Dis3 depleted by addition of thiamine for > 12h.

B MS analysis of a comparative poly(A)+ RNA interactome capture experiment for the nuclear exosome mutant *rrp6Δ*. In the volcano plot, p-values (−log, two-sided moderated Student's t-test) are plotted against the fold change of mean MS intensities (log_2) of proteins recovered from the oligo(dT) pull-downs of UV-crosslinked samples (3 J/cm_2) for wild type and *rrp6Δ* (n = 3 biological replicates). Data from [9], ProteomeXchange accession PXD016741. CF1A components are enriched on poly(A)+ RNA in the exosome mutant. Source data are provided as a Source Data file.

C Enrichment of CPAC components, various splicing factors, and representative RNAPII and nucleosome components on poly(A)+ RNA in the nuclear exosome mutants *mtl1-1* and *rrp6Δ* based on the MS analysis of comparative poly(A)+ RNA interactome capture experiments as in B. Colour indicates the log_2 fold change (mutant over wild type), circle size the p-value (moderated Student's t-test). Data from [9], ProteomeXchange accession PXD016741; cells were grown in EMMG and labelled with 4-thiouracil for 4.5h prior to crosslinking. Source data are provided as a Source Data file.

Supplementary References

1. Elfmann, C. & Stülke, J. PAE viewer: a webserver for the interactive visualization of the predicted aligned error for multimer structure predictions and crosslinks. *Nucleic Acids Res* **51**, W404–W410 (2023).

2. Evans, R. *et al.* Protein complex prediction with AlphaFold-Multimer. *bioRxiv* 2021.10.04.463034 (2022) doi:10.1101/2021.10.04.463034.

3. Chen, H.-M., Futcher, B. & Leatherwood, J. The fission yeast RNA binding protein Mmi1 regulates meiotic genes by controlling intron specific splicing and polyadenylation coupled RNA turnover. *PLoS One* **6**, e26804 (2011).

4. Martín Caballero, L. *et al.* The inner nuclear membrane protein Lem2 coordinates RNA degradation at the nuclear periphery. *Nature Structural & Molecular Biology 2022 29:9* **29**, 910–921 (2022).

5. Atkinson, S. R. *et al.* Long noncoding RNA repertoire and targeting by nuclear exosome, cytoplasmic exonuclease, and RNAi in fission yeast. *RNA* **24**, 1195–1213 (2018).

6. Kilchert, C. *et al.* Regulation of mRNA levels by decay-promoting introns that recruit the exosome specificity factor Mmi1. *Cell Rep* **13**, 2504–2515 (2015).

7. Birot, A. *et al.* RNA-binding protein Mub1 and the nuclear RNA exosome act to fine-tune environmental stress response. *Life Sci Alliance* **5**, e202101111 (2021).

8. Lemay, J.-F. *et al.* The RNA exosome promotes transcription termination of backtracked RNA polymerase II. *Nat Struct Mol Biol* **21**, 919–926 (2014).

9. Kilchert, C. *et al.* System-wide analyses of the fission yeast poly(A)+RNA interactome reveal insights into organization and function of RNA-protein complexes. *Genome Res* **30**, 1012–1026 (2020).
