# Supplementary material for: DEAD-box ATPase Dbp2 is the key enzyme in an mRNP assembly checkpoint at the 3’-end of genes and involved in the recycling of cleavage factors
Source: Nat Commun. 2024 Aug 9;15:6829. doi: 10.1038/s41467-024-51035-z (PMC11315920; doi:10.1038/s41467-024-51035-z)
Supplement: Supplementary file 3 — Description of Additional Supplementary Files [file 41467_2024_51035_MOESM3_ESM.pdf]

Description of Additional Supplementary Information

File Name: Supplementary Data 1-5

Description: Strains, oligonucleotides, plasmids, antibodies and MaxQuant parameters used in this study

File Name: Supplementary Software 1

Description: Semiautomated ImageJ macro script for quantitation of microscopy data
